# Exploring the Therapeutic Affordances of Self-Harm Online Support Communities: An Online Survey of Members

**DOI:** 10.2196/mental.8084

**Published:** 2017-10-13

**Authors:** Neil S Coulson, Emma Bullock, Karen Rodham

**Affiliations:** ^1^ University of Nottingham Nottingham United Kingdom; ^2^ Staffordshire University Stoke-on-Trent United Kingdom

**Keywords:** self-harm, social network, social support, qualitative research, online support group

## Abstract

**Background:**

A growing number of online communities have been established to support those who self-harm. However, little is known about the therapeutic affordances arising from engagement with these communities and resulting outcomes.

**Objective:**

The aim of this study was to explore the presence of therapeutic affordances as reported by members of self-harm online support communities.

**Methods:**

In total, 94 respondents (aged 13-63 years, mean=23.5 years; 94% female) completed an online survey exploring their experiences of engaging with a self-harm online support community. Respondents varied in terms of how long they had been accessing an online community, with 22% (21/94) accessing less than 1 year, 39% (37/94) 1 to 2 years, 14% (13/94) 2 to 3 years, and 24.5% (23/94) more than 3 years. Responses were analyzed using deductive thematic analysis.

**Results:**

The results of our analysis describe each of the five therapeutic affordances that were present in the data, namely (1) *connection*, the ability to make contact with others who self-harm for the purposes of mutual support and in so doing reduce feelings of loneliness and isolation; (2) *adaptation*, that is, how use of online support varies in relation to the personal circumstances of the individual user; (3) *exploration*, that is, the ability to learn about self-harm and learn about strategies to reduce or stop self-harming behavior; (4) *narration*, that is, the ability to share experiences, as well as read about the experiences of others; and (5) *self-presentation*, that is, how and what users present about themselves to others in the online community.

**Conclusions:**

Our findings suggest that engagement with self-harm online support communities may confer a range of therapeutic benefits for some users, which may serve to minimize the psychosocial burden of self-harm and promote positive coping strategies. In addition, the online nature of the support available may be helpful to those who are unable to access face-to-face support.

## Introduction

### Background

Regardless of suicidal intent or other motivations, self-harm is a term used to describe all nonfatal acts of intentional self-injury or self-poisoning [1]. In addition to being a significant risk factor for completed suicide [2], it is also associated with elevated all-cause mortality [3]. Self-harm is also linked to poorer psychosocial outcomes including depression, anxiety, and substance use [4] and carries with it considerable health services and social costs [5]. Self-harm appears to be more common in females than males, though this gap has narrowed in recent years [6] and appears to further diminish across the lifespan [7]. It tends to be more prevalent in younger age groups [8]. Among females, rates of self-harm appear to be highest in the age group of 15 to 24 years, but for males this tends to be in their late twenties and early thirties. For older age groups, self-harm appears to be less prevalent but does appear to be related to higher levels of suicidal intent [6].

Internet use has increased globally by 933.8% from June 2000 to March 2017, resulting in approximately 49.6% of the world’s population being online [9]. While usage of the Internet continues to increase across all age groups and both genders [10,11], young people aged 16 to 24 years remain the highest users, with 99.2% in the United Kingdom accessing the Internet in the previous 3 months [10] and 96% of young people aged 16 to 29 years having used the Internet in the United States [11]. With the emergence and burgeoning of Internet use, the way in which some individuals communicate their self-harm experiences has changed. Before the Internet, any disclosure around self-harm was restricted to face-to-face networks (eg, friends and family), telephone support lines (eg, Samaritans), or health professionals. Nowadays, it is becoming more common for experiences of self-harm to be shared virtually via photographs, videos, and online discussions [12-16]. Although much of the work exploring Internet use has reported negative effects, including triggering as well as normalizing self-harm [13,14,17], sharing of self-harm methods [18-20], and methods to conceal self-harm from others [13,20], other researchers have demonstrated the positive impact that the Internet can have. For example, Baker and Fortune [21] reported that participants felt that self-harm and suicide sites had contributed to their recovery and facilitated change *better than any therapy.*

As a result of technological advances, there is now a greater opportunity for individuals who self-harm to interact with each other online. In particular, there are a growing number of online support communities (also known as *online support groups*) that have been established to support those who self-harm, and these have typically been developed using asynchronous discussion forum platforms and social networking sites (eg, Facebook). These online support communities offer new opportunities to those who self-harm to obtain information, advice, and support [16]. Furthermore, they enable the connection of otherwise isolated individuals [13,14,22] who can receive support from like-minded individuals, feel less isolated, and find a community that understands their self-harm with whom they can discuss topics of mutual interest [23-25].

Thus, there is a small but growing body of literature that has explored the use of self-harm online support communities, notably discussion forums, and reported both positive and negative consequences [26]. However, the bulk of previous studies that considered self-harm online support communities have been largely descriptive, with no underpinning theory that explores the relationship between users’ online behavior and reported outcomes. It is our contention that to advance our knowledge and understanding of the role of online support communities for those who self-harm, there needs to be a greater emphasis placed on understanding the interaction between the individual who uses the online community and the specific functionality afforded by its underpinning platform and how this relates to health outcomes. Therefore, to explore how engagement with self-harm online support communities may impact on users, our study considered the perceived therapeutic affordances of such interactions.

### Affordance Theory

The roots of affordance theory can be traced back to perceptual and cognitive psychology and are based on how individuals perceive the objects around them in the environment. That is, what the specific object is and what potential use it affords [27]. The properties of any specific object will therefore contribute to its perceived affordance as will the varying experiences, beliefs, and goals of an individual. What is central to this theory is the interaction between the individual and the object and its subsequent outcomes. Therapeutic affordances have been described as the *actionable possibilities* of the object as determined by the individual [28], and in this instance, the object of our study is an online support community. Therefore, by focusing on the therapeutic affordances conferred by online support communities, we can consider not only their use but also their impact.

**Figure 1:**
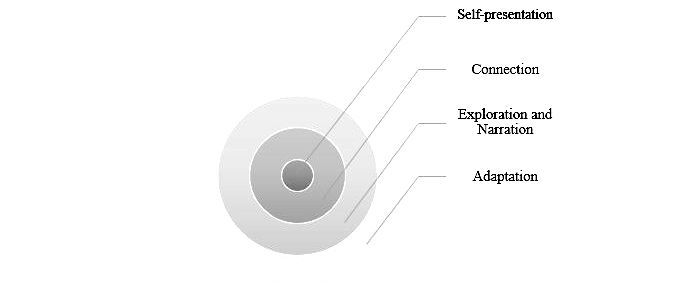
SCENA model (Self-presentation, Connection, Exploration, Narration, and Adaptation) of therapeutic affordance of social media (adapted from Merolli et al, 2014).

The utility of affordance theory can be illustrated by the work of Merolli et al [28]. In a global survey of social media use by patients living with chronic pain, five main therapeutic affordances arising from social media were identified and described: *self-presentation* (ie, the level of information presented to the world via social media), *connection* (ie, the use of social media to reach out to others in similar situations, share or exchange information, and offer support), *exploration* (ie, the use of social media for guidance toward useful information), *narration* (ie, sharing experiences via social media), and *adaptation* (ie, the way social media enabled respondents to adapt their self-management behaviors in relation to their condition status and needs at particular points in time in various ways). These affordances were then used to develop the SCENA model (ie, Self-presentation, Connection, Exploration, Narration, and Adaptation) that is depicted in Figure 1. Merolli et al (2014) propose that at the core of this model are preferences and perceptions relating to one’s image or digital identity. *Self-presentation* will then feed into the ability of social media to *connect* individuals. The next layer, they propose is shared by both *exploration* and *narration*, both of which acknowledge varying preferences for self-presentation and how individuals connect. The outer layer in this model depicts *adaptation*, which reflects how social media can be used for self-management behaviors as and when the need arises at different points in time. This, they argue, will influence and be influenced by affordances to varying degrees.

Until now, the potential therapeutic affordances that may be conferred through engagement with self-harm online support communities has not been the focus of investigation. Therefore, the aim of this study was to explore the presence of therapeutic affordances arising from engagement with self-harm online support communities as reported by those individuals who engage with them; specifically, to consider the relationship between any identified therapeutic affordances and subsequent outcomes. To achieve this, our work was guided by Merolli et al (2014) SCENA model and used as a theoretical framework through which to consider and reflect upon the experiences of those who engage with self-harm online communities.

## Methods

### Recruitment and Data Collection

This study involved the recruitment of individuals who access self-harm online support communities. To identify potential communities, a search using Google was undertaken incorporating a range of keywords in various combinations, including “self-harm,” “self-injury,” “online support groups,” “support,” “message boards,” and “forums.” The results of this search yielded nine self-harm online support communities that were deemed eligible to be contacted. Our inclusion criteria included (1) user-led community, (2) active with at least 30+ posts per month, (3) moderators could be identified with contact details available, (4) the terms and conditions of the community did not preclude research-related activity, and (5) English language. Following contact with moderators from each community, positive responses were received from three communities (two asynchronous forums and one Facebook community). The communities that were willing to assist with our study were based in either the United Kingdom (one forum with 1000+ members and the Facebook community with 30,000+ followers) or North America (one forum with 1000+ members). It should be noted that since the time of data collection, the North American forum has since closed. The remaining communities which were contacted either declined the invitation (N=2; both asynchronous forums) or failed to respond (N=4; three forums and one Facebook group). For the two forums that declined to take part, no explanation was provided.

For those communities granting permission, a recruitment message was posted to their discussion forum outlining the aims of the study and inviting interested members to click on a link to the online survey hosted by Bristol Online Surveys. Upon arrival at the landing page, members were provided with additional information concerning the study and were then asked to complete an online consent form. Following this, members completed some background questions (age, gender, and country of residence) and their self-reported use of self-harm online support communities. Next, they were invited to respond to a set of open-ended questions that explored their motives and experiences of using online support communities, including perceived benefits or problems (see Textbox 1). The questions used in our survey were based on those very successfully used in previous research, though no direct piloting of these questions with the target group took place. Within the online survey, each question was followed by an expanding text box, which meant participant responses were not limited by space.

**Open-ended questions used in the online survey.**

- Why did you decide to become a member of an online support group?
- Has being a member of the group helped you in any way? If so, please give some examples.
- What do you feel are the benefits of taking part in an online support group?
- Have you encountered any problems while being a member of an online support group?
- Has being a member of an online support group had an impact on any of your offline relationships?

### Ethical Considerations

Before the commencement of data collection, the research protocol was considered and approved by the institutional ethics review committee of the University of Nottingham. As per accepted ethical practice [29], our online survey was prefaced with a comprehensive information page that outlined the nature of the study, rights as a research participant, withdrawal procedures, together with contact details of the research team. After considering this information, respondents were then directed to an online consent page which required them to select “yes” in response to a series of consent statements, all with the option “yes or no.” To ensure that data could be retrieved in the event of a query or a request to withdraw their data, all respondents were asked to create a unique password and quote this password in any correspondence with the research team. Ultimately, no respondent chose to retrospectively withdraw their data.

### Participants

In total, 94 online support group members responded to our open-ended questions. Ages ranged from 13 to 63 years, with a mean age of 23.5 years and the majority (88/94, 94%) being female. In terms of country of residence, 51 % (48/94) were from the United Kingdom, 19% (18/94) from North America, and 7% (7/94) from Australia, with remainder from other European countries (6% 6/94), South America (1/94, 1%), and Asia (5/94, 5%), with 10% (9/94) choosing not to report. Respondents varied in terms of how long they had been accessing an online support community, with 22% (21/94) less than 1 year, 39% (37/94) 1 to 2 years, 14% (13/94) 2 to 3 years, and 24.5% (24.5) more than 3 years.

### Data Analysis

Responses to the open-ended questions were analyzed by the first author (NC). Initially, all responses were analyzed using a deductive approach (see Table 1 for coding framework), seeking evidence of the presence of the therapeutic affordances outlined by Merolli et al [28] in conjunction with the guidelines set out by Braun and Clarke [30]. In the first instance, each set of survey responses were read and reread several times to become familiar with the data. Next, interesting and salient features and patterns within the data were coded, using the language of the respondents wherever possible. From this, codes were then arranged into meaningful groups to form potential subthemes for each affordance. In cases where codes appeared throughout the data, these were considered as potential themes. All data relevant to each potential theme were gathered together, and then each theme was reviewed, refined, and then allocated a clear definition and label. In addition, reviewing the language typically used in these themes allowed them to be organized into the final set of therapeutic affordances. To check that the themes identified reflected the data, the second and third authors reviewed the survey responses and together with the first author confirmed the final set of themes.

**Table 1:** Coding framework for deductive thematic analysis with illustrative quotes.

Therapeutic affordance	Definition and illustrative examples
Connection	An ability to connect to others in similar situations; the sharing or exchange of information; offers of support
	“...people who suffer with similar disorders understand better.”
Exploration	An ability to search for information or advice about self-harm; learning about self-harm, including causes; coping and self-management strategies; sources of support
	“...reading and learning information about self-harm.”
Narration	An ability to share personal experiences, opinions, and viewpoints; accessing other people’s experiences
	“...writing my thoughts to a forum where other people can see them, and read other people’s posts has been very helpful.”
Self-presentation	An ability to control or exercise autonomy over information disclosure online; discussion of private versus public nature of online activity
	“...the anonymity helps when I want to open up without freaking people out.”
Adaptation	An ability to engage with an online support community depending on individual needs, circumstances, priorities, and health status.
	“I was recovering from years of self harm. Recently, I’ve been feeling urges again.”

## Results

### Engagement With the Survey

Overall, the number of community members responding to each open-ended question (see Textbox 1) was as follows: Q1=91/94, Q2=86/94, Q3=84/94, Q4=78/94, and Q5=70/94. In addition, the amount of text written by respondents ranged from 2 to 78 words (mean=16.12) for Q1, 1 to 77 words (mean=20.23) for Q2, 1 to 112 words (mean=17.2) for Q3, 1 to 122 words (mean=10.5) for Q4, and 1 to 54 words (mean=10.01) for Q5.

### Findings From the Analysis of Open-Ended Survey Questions

Our deductive thematic analysis described each of the five therapeutic affordances and related outcomes that were present, to varying degrees, in the data (see Table 2). The most frequently mentioned theme, as determined by the number of individuals commenting, was that of *connection* (83/94, 88%) and *adaptation* (48/94, 51%), followed by *exploration* (44/94, 47%) and *narration* (43/94, 46%), and finally *self-presentation* (37/94, 39%).

**Table 2:** Therapeutic affordances, processes, and outcomes (+ or −).

Therapeutic affordance	Process	Outcome (positive + or negative −)
Self-presentation	Autonomy	Identity (+ and −)
	Disclosure	
Connection	Interaction	Reduced isolation (+)
	Mutual support	Supportive relationships (+)
		Interpersonal conflict (−)
Exploration	Information-seeking	Knowledge (+)
	Learning	Adaptive coping strategies (+)
Narration	Sharing experiences	Understanding (+)
	Emotional catharsis	
Adaptation	Personal circumstances	Availability (+)

#### Self-Presentation

At the heart of this affordance was the ability to exercise autonomy over the discussion of self-harm and the disclosure of personal information and experiences, as well as a clear preference for anonymous online interactions to protect their identity and retain privacy.

Several of our respondents commented on the value they placed on being able to reveal aspects of themselves to fellow members of the community. In particular, being able to discuss self-harm with “strangers” rather than to family, friends, and wider face-to-face networks was welcomed:

Sometimes it is easier to open up to a stranger than to a person you know your whole life.

However, the benefits derived from the online communities in terms of self-presentation also included how they revealed aspects of themselves, for example, one respondent stated:

It’s sometimes easier to type how you feel than speak it...

Several comments were made describing how they were able to present themselves in a more “honest” or truthful way. What appeared to permeate many of the comments made was the view that traditional face-to-face networks restricted discussion of self-harm, but the online nature of the support communities overcame this and provided new opportunities for their self-harming to be acknowledged and discussed. It was evident in many responses that the discussion of self-harm was for some individuals a new and much needed opportunity, arising directly from their decision to join the online community.

The clear preference for anonymous interactions was evident across many comments made by our respondents. Indeed, the ability to reveal aspects of their identity online appeared to be closely linked to the perceived anonymity that was conferred:

Being anonymous. You can talk about what’s going on with you and nobody in your life will find out. It’s a beautiful thing

... *the anonymity helps when I want to open up without freaking people out.*

However, apprehension was evident in some respondents as they expressed concerns around engaging with the Facebook group for fear their self-harming behavior would be revealed to others:

I didn’t even like the Facebook page because I was afraid someone might suspect something if they found me liking a self-harm related page.

I didn’t like the page, because others can see which pages you like.

#### Connection

This affordance focused on the ability to connect with others, and respondents used this connection to support each other, to overcome feelings of isolation, and develop supportive relationships. However, at times there appeared to be instances of conflict between members and situations where outsiders would deliberately try to cause harm to community members.

Many of our respondents commented on the fact that through accessing an online support community, they could interact with other people who also self-harmed. On several instances respondents discussed the importance of seeking out and connecting with others because “people who suffer with similar disorders understand better.” Indeed, this notion of understanding resonated across many comments made as respondents described their hopes that by joining an online community they would find others who could “relate,” “listen,” and that were facing “similar struggles.”

A common experience described by respondents was that of feeling “totally alone” with a “lack of support available.” For some, this reflected the fact that their self-harming was done in secret, and this made it difficult to “reach out” and find support from people who would understand:

I self-harmed in secret and felt totally alone. I wanted to stop and needed support to try but no one in real life knew I was even unhappy.

For others, there was nobody in the “real world” who could provide this support, typically because they would “not understand,” and there was a widespread fear that they would be ridiculed:

I’m scared of other people making jokes of me.

A widespread theme within respondents’ answers was that of “mutual support” and the potential of the online community to bring people together:

It’s great having people to encourage you and understand you. When you have a bad day you can have the support you need, whether that’s a pep-talk or sympathy, or just an ear to listen. It’s great because everyone knows a bit about what you’re going through.

In particular, the ability to connect with others online appeared to provide respondents with a sense of belonging:

It makes you feel like you’re part of something greater like people are almost united by their illness.

Indeed, this notion of connection was evident across several respondents’ comments particularly as they described the benefits of “having someone to talk to, who knows what you’re going through and can relate.” A number of comments were made which described how this online support was provided in a way that was nonjudgmental and risk free. As one respondent described:

You get to tell your story and be honest about how you feel knowing that you will not be judged.

Through this connection with similar others, many respondents described how being part of an online support community had reduced their sense of isolation and loneliness and helped them feel less alone:

The greatest benefit is the feeling of not having to feel alone in your issues.

Indeed, the word “alone” was used by several respondents, particularly as they described how it felt to self-harm but to keep it hidden from friends and family. As a result of finding others who are in the same position, several respondents described how they were coming to accept the reality of their position:

It helped me to realize that I’m not strange.

The group has helped me to see that I am not alone, even in the most bizarre behaviours.

It was evident from the responses provided that the connections with others made through the online support communities were meaningful, with some describing the “supportive relationships” that had become established. Respondents discussed their “being part of a community” and the “friendship” that engagement with the online support community brings. One respondent emphasized the role of the online support community in facilitating connections with similar others:

It makes you feel like you are part of something greater like you are almost united by their illness.

Despite this, connecting with others through an online support community was not always a positive experience. For example, respondents described situations in which there existed some conflict, either through a difference of opinion or through deliberate intent to cause trouble:

People passionately disagree at times which can sometimes cause tension

I think there are always going to be the idiots that like to try and start fights.

#### Exploration

At the heart of the “Exploration” therapeutic affordance was the ability to seek information, learn and acquire knowledge about self-harm, and the impact of this on the development of adaptive coping strategies.

Respondents described how their online support community was helpful in terms of “reading and learning information about self-harm” and a place where they could “ask questions” and get answers. The community was viewed as a valuable repository of information which could be accessed at any point:

There many times I’ve just needed some info and there’s plenty on...[name of community].

For some, the online support community provided a new opportunity to seek information and advice. As one member explained:

I wanted to know more about it, because during endeavours to learn more in years past, information was scarce.

Through the online community, respondents could learn more about the commonly experienced thoughts and “urges.” As one member explains, the online community helped them “get answers about why I had certain feelings.”

As well as learning more about self-harm generally, respondents described how they obtained practical advice on strategies to cope with and manage their self-harm behavior. As a consequence, several respondents described how they were then able to implement new adaptive coping strategies to combat urges to self-harm:

I’ve read a lot on this site and have found ways to distract myself when I feel triggered.

Other comments made by fellow respondents also demonstrated how engagement with the online community positively impacted on how they managed their ongoing struggles:

It has enabled me to find other, less damaging coping methods.

...given me ideas about how to manage it.

...also given me practical advice on coping techniques, first aid and other things.

For some, the ability to understand the nature of self-harm and how to manage it had yielded positive outcomes. As one respondent explained:

Yes, it has stopped me from self-harming and the pictures they boost often boost my self-esteem.

Others also confirmed that they now “cut less” or had fewer urges to do so.

#### Narration

Respondents described how they shared their own experiences of self-harm, as well as hearing about the experiences of others through the online community. Through this narration, and that of others, respondents described a range of positive benefits, but some problems were also identified (ie, triggering content being posted online).

Through narrating their self-harm experiences online, several respondents noted the positive impact. For example:

...writing my thoughts to a forum where other people can see them, and read other people’s posts has been very helpful.

Others noted how writing about their experiences online helped them to organize their thoughts and communicate more effectively:

Writing things out can help to get them out my head and make them clearer.

In contrast, respondents also explained how in some situations reading posts by other community members could be unhelpful. As one respondent explained:

At my lowest I would compare my problems to others and think they were not important.

In other instances, respondents provided examples of why messages posted by others could be distressing:

Sometimes it is hard seeing people who say they want to die.

Some respondents describe altogether more serious problems when the content of messages posted by other community members appeared to “trigger” difficult thoughts, feelings, and behavior:

There have been occasions when members have posted either triggering words or pictures, which have triggered me to feel low or hurt myself.

#### Adaptation

At the heart of this affordance were the personal circumstances of the individual users and how these were related to engagement with the online community. This affordance reflects both the varying circumstances at the point of deciding to join a self-harm online community, as well as their subsequent and ongoing engagement with each community.

In explaining the decision to join a self-harm online community, many respondents described either the absence of or barriers to accessing face-to-face support for their self-harming behavior:

I needed somewhere to go and talk about my issues that I couldn’t talk about with family and friends.

I wasn’t receiving any useful help from the NHS [National Health Service] and was on a stupidly long waiting list. I need some support...

In particular, feelings of guilt, shame, or embarrassment were a significant component of their view that face-to-face support was not an option, but online support could be a useful alternative. As one respondent explained:

I am confused and baffled by my behaviour and feel a deep sense of shame and embarrassment and loneliness. I hoped this community might help.

Specifically, notions of privacy and anonymity were salient across many comments, and these were important considerations in their decisions to engage with a self-harm online community.

In other instances, respondents described changes in their self-harm behavior, typically deterioration in their mental health and well-being and/or an escalation of their urges and attempts to self-harm:

Because my self-harm acts were getting out of control.

I was recovering from years of self-harm. Recently, I’ve been feeling urges again.

Many comments made by the respondents illustrated how they visited and revisited the community for support during critical periods when they were “in crisis” or when they were “falling back into self-injury.” Others considered how their mood was related to their use of the community:

I’ve managed to stop for a couple of weeks and when I felt low I could talk to people on the ground to distract and advise you.

What was evident across the responses was the fact that engagement with the support community was related to how they were feeling about their self-harm. For some, the challenges were during periods when other sources of support could not be accessed, and so, the 24-hour nature of the community was considered helpful:

It has helped by being 24 hr because I mostly get sad at night so I can’t call my consuler [counsellor].

## Discussion

### Principal Findings

The aim of this study was to explore the therapeutic affordances that may be conferred through engagement with self-harm online support communities, as described by those individuals who use them. We based our deductive thematic analysis on the therapeutic affordances described by Merolli et al (2014), and our findings offer additional support for the validity of these affordances in this group. Our findings describe each of the five therapeutic affordances in the context of engagement with self-harm online support communities.

*Connection* was the most commonly described therapeutic affordance by our respondents, with emphasis being placed on the benefits of interacting with others who share similar experiences. Previous research within the self-harm literature has pointed to the potential benefits of having support [17,20,31] but has noted that obtaining this support may be difficult, for various reasons including stigma, shame, or embarrassment [32]. This might therefore account for the large number of respondents who noted *connection*, as engaging with an online support community may be one convenient and anonymous way to access support. Furthermore, many respondents described feelings of isolation and loneliness, particularly as they often kept their self-harming behavior hidden from public view. The lack of a supportive face-to-face network may heighten feelings of isolation, and so, connecting with similar others online may provide new opportunities for much needed support.

Although previous work has not always identified the affordance *adaptation* [33], this study did find that several respondents explicitly discussed how their use of an online support community varied depending on how they were feeling and/or current situation with regards self-harm urges or behavior. However, on reading across the responses to our survey questions, it could be argued that use of a self-harm online support community is crucially linked to respondents’ needs, current feeling, and general sense of well-being. As a result, many respondents may have used the online community to address a specific need, but this did not necessarily translate into any explicit comment in their responses to our open-ended survey questions.

The affordance of *exploration* was also frequently commented upon by our respondents as they described the ability to find information, ask questions, and gain knowledge about self-harm online. Of importance was the opportunity to find practical advice, which could be used to prevent further self-harm and to help them implement adaptive coping strategies.

In terms of *narration*, the online support community was viewed as a safe place to share stories and experiences, as well as to provide information and advice. The importance of experiential information and advice has been noted elsewhere in the literature [34-37], and the results of our study suggest that the online support community may be a useful venue through which experiences can be shared.

The affordance *self-presentation* was considered valuable to our respondents in relation to the perceived privacy and anonymity, whereas the community conferred. Again, other work in the field of self-harm has confirmed the importance of safe spaces for individuals to freely disclose information and details about their self-harm history and ongoing struggles. Our findings suggest that features of the online community (ie, restricted access and anonymity) may be viewed as particularly helpful in supporting individuals as they open up to others online. Interestingly, some differences were noted between the users of the Facebook group and those using the forums in relation to concerns about privacy.

Despite several positive benefits being discussed, there were some concerns expressed by respondents about their online experiences. By far, the most problematic aspect centered on the content posted by other community members and its potential to trigger self-harm behavior. However, it was noted by several respondents that warnings were helpful, and so, this practice may be encouraged going forward. Other lesser concerns focused on the interactions between members and the fact that sometimes arguments can take place. To limit the impact of these episodes, moderators may usefully step in to restrict content which may cause upset to other members. Finally, some respondents commented upon the existence of people who seemed determined to cause trouble within an online community. Again, the role of the moderator may be crucial in limiting any potential damage to the community dynamics, as well as safeguarding the welfare of individual members.

To date, various theoretical frameworks have been employed to assist in our understanding of how users engage with online support communities and what impact this engagement may have on their experience of illness and psycho-social well-being. One common example is that of social support, and previous studies adopting this theoretical framework have shed light on the potential health-related benefits of engagement [38]. While there is arguably some conceptual overlap between the various therapeutic affordances and social support, social support has commonly been applied from a health outcome-oriented perspective [39]. It is our belief that as a result of the proliferation in the types of platforms being used for online peer-to-peer support, it is now timely that we adopt a theoretical framework which explicitly acknowledges the interaction between the user, the functionality of the platform, and any resulting outcomes. With this in mind, we considered affordance theory [27] and found this framework to be helpful while interpreting the responses to the open-ended questions posed in our survey. This approach was especially relevant when differences between the affordances across the two platforms represented in the study were recorded (eg, differences in the self-presentation affordance). That said, we believe there are several research avenues to explore going forward, not least the role of individual factors and/or illness-specific factors and how these relate to the engagement with self-harm online support communities and how varying levels of engagement may relate to health outcomes. However, for now, we believe that adoption of this framework to guide our deductive analysis proved beneficial in understanding how people experiencing self-harm may use online support communities.

### Limitations of the Study

There are several limitations to this study that should be acknowledged. First, it is not clear how representative the sample is of the self-harm population. Although the mean age of our participants (23.5 years old) is broadly consistent with prevalence studies [6], our sample is heavily skewed toward females. Whereas the bias toward female respondents is both consistent with previous studies of online support group use for conditions that affect both genders [35], it does leave the male *voice* somewhat unheard, and therefore, future work should actively seek to redress this and employ specific strategies to ensure sufficient representation from males. Second, despite receiving positive responses from three online support communities, we were not able to access the other six communities which we approached to take part in the study. Therefore, it remains unclear whether our results may have been different if we were able to access these additional communities. The specific focus and dynamics of these (and other) communities may differ from those included in this study, and therefore, these groups may have offered different opportunities and ultimately different affordances. Finally, we employed an online survey methodology to collect data from members of self-harm online support communities. The decision to use an online survey format was felt to sit comfortably with the fact that the focus of the study is on people’s experiences of asynchronous text-based support. In addition, it was evident from the responses provided that the online survey approach conferred a degree of anonymity which was felt important, particularly to those who may not have disclosed their self-harming behavior to their social networks. In addition, we did not observe any responses, which may suggest that our respondents were answering anything other than honestly. That said, it remains to be seen whether an alternative format may have elicited more detailed, longer, and richer insights into their online experiences. We have reported some basic descriptive analysis of engagement with our survey questions, and although this data is encouraging, it is pertinent to at least acknowledge the potential limitations of this static open-text online survey. In addition, we did not have the opportunity to pilot our survey questions, and future research should ensure that this is undertaken.

### Conclusions

Our survey findings suggest that engagement with self-harm online support communities may confer a range of therapeutic benefits for some users, which in turn may serve to minimize the burden of illness. Furthermore, self-harm online support communities may serve as a useful public health intervention through which individuals experiencing a range of negative impacts may engage in anonymous mutual support in ways that foster individual adaptive coping strategies and improve psychosocial well-being.

## Abbreviations

SCENA: Self-presentation, Connection, Exploration, Narration, and Adaptation
